# Energy Potential of Biomass from Conservation Grasslands in Minnesota, USA

**DOI:** 10.1371/journal.pone.0061209

**Published:** 2013-04-05

**Authors:** Jacob M. Jungers, Joseph E. Fargione, Craig C. Sheaffer, Donald L. Wyse, Clarence Lehman

**Affiliations:** 1 Conservation Biology Graduate Program, University of Minnesota, Saint Paul, Minnesota, United States of America; 2 The Nature Conservancy, Minneapolis, Minnesota, United States of America; 3 Department of Agronomy and Plant Genetics, University of Minnesota, Saint Paul, Minnesota, United States of America; 4 College of Biological Sciences, University of Minnesota, Saint Paul, Minnesota, United States of America; University of Leipzig, Germany

## Abstract

Perennial biomass from grasslands managed for conservation of soil and biodiversity can be harvested for bioenergy. Until now, the quantity and quality of harvestable biomass from conservation grasslands in Minnesota, USA, was not known, and the factors that affect bioenergy potential from these systems have not been identified. We measured biomass yield, theoretical ethanol conversion efficiency, and plant tissue nitrogen (N) as metrics of bioenergy potential from mixed-species conservation grasslands harvested with commercial-scale equipment. With three years of data, we used mixed-effects models to determine factors that influence bioenergy potential. Sixty conservation grassland plots, each about 8 ha in size, were distributed among three locations in Minnesota. Harvest treatments were applied annually in autumn as a completely randomized block design. Biomass yield ranged from 0.5 to 5.7 Mg ha^−1^. May precipitation increased biomass yield while precipitation in all other growing season months showed no affect. Averaged across all locations and years, theoretical ethanol conversion efficiency was 450 l Mg^−1^ and the concentration of plant N was 7.1 g kg^−1^, both similar to dedicated herbaceous bioenergy crops such as switchgrass. Biomass yield did not decline in the second or third year of harvest. Across years, biomass yields fluctuated 23% around the average. Surprisingly, forb cover was a better predictor of biomass yield than warm-season grass with a positive correlation with biomass yield in the south and a negative correlation at other locations. Variation in land ethanol yield was almost exclusively due to variation in biomass yield rather than biomass quality; therefore, efforts to increase biomass yield might be more economical than altering biomass composition when managing conservation grasslands for ethanol production. Our measurements of bioenergy potential, and the factors that control it, can serve as parameters for assessing the economic viability of harvesting conservation grasslands for bioenergy.

## Introduction

Perennial biomass is an alternative to conventional starch-based biofuel feedstocks such as corn. It may improve land-use efficiency, reduce greenhouse gas emissions, promote biodiversity, and support other components of sustainability [1]–[3]. Research comparing ecosystem services of various native and non-native perennial bioenergy crops in the Upper Midwest indicates that bioenergy systems with more plant species support greater avian diversity [4], abundance and diversity of beneficial arthropods [5], carbon storage and complexity of belowground food webs [6]. In many regions of North America, diverse grasslands have not produced as much gross biomass as dedicated energy crops grown in monoculture such as switchgrass [7]. This has initiated questions regarding the economic viability of diverse grassland bioenergy, yet few studies have quantified bioenergy yields from diverse perennial plantings over multiple years. Only recently have studies compared the bioenergy potential of mixed-species grasslands harvested with production-scale techniques in various regions of the Upper Midwest [8].

Growing biomass on land unsuitable for commodity crops transforms the economic outlook for bioenergy systems. Bioenergy production from feedstocks grown on marginal or underutilized land, such as land enrolled in the Conservation Reserve Program (CRP), can provide immediate greenhouse gas benefits [9] while avoiding competition for land between food and energy crops [10]. One idea is to harvest biomass from CRP land as revenue to supplement government subsidies, potentially incentivizing renewal of CRP contracts and offsetting recent trends in expiring CRP acreage [11]. Current CRP regulations do not allow biomass harvest from land enrolled in the program. If economic opportunities from bioenergy initiate new regulations that allow biomass harvest, these regulations should be designed to support the original intentions of the CRP, including improved wildlife abundance [12], an important component of biodiversity.

Other conservation lands managed for wildlife by state, federal, and non-profit agencies have been planted with mixtures of perennial grassland species. These may serve as biomass sources for energy production. Studies are underway to determine the effects of biomass harvest on resident wildlife in various types of conservation grasslands [13]. If research concludes that conservation grasslands can be managed for bioenergy and biodiversity simultaneously, then the quality and quantity of harvested biomass from conservation lands should be considered before bioenergy management is implemented.

The amount of bioenergy from conservation grasslands depends on both biomass quantity and quality. One means of measuring biomass quantity is to multiply yields from CRP fields in different regions of North America by estimates of available acreage [8], [14]–[16]. These yields can then be extrapolated to estimate biomass from land not currently enrolled in, but eligible for conservation programs. Another important component of predicting bioenergy potential is biomass quality, often defined by the mineral and sugar concentrations of the biomass. Mineral concentrations are used to predict conversion efficiency for thermochemical energy production. High concentrations of alkali metals in post-combustion ash lead to slagging and fouling in thermochemical systems [17], while high concentrations of N, S, and other elements pose issues of oxide emissions and possibly nutrient removal from soils in long-term harvested systems [18]. Predicting the efficiency of biofuel production with biochemical technologies requires measuring the plant sugar and carbohydrate concentrations. High values of cellulose and hemicellulose relative to lignin results in greater liquid biofuel potential [19].

Variation in the quantity and quality of grassland biomass with respect to energy production–hereafter called bioenergy potential–can occur due to variation in plant species composition, geographic location, and management activities. Plant composition influences bioenergy potential with studies indicating positive relationships between (i) biomass yield and planted species richness [2] and (ii) relative cover of warm-season grasses (C4) and lignocellulose ratios that favor ethanol production [14]. In southern Iowa, spatial variation in biomass yield and elemental composition was greater within fields than between fields and was correlated to individual species within cool-season (C3) grasslands [20]. A broad-scale analysis of switchgrass yields across the Great Plains indicated that within-field variation is small enough to consider the mean biomass yield of a field for modeling purposes [21]. Di Virgilio *et al*. found correlations between switchgrass yields and both soil fertility and moisture, which were interpreted as sources of within-field variation [22].

Management activities, including harvest, also affect bioenergy potential. Harvesting biomass after senescence allows for plants to translocate nutrients to belowground tissues, but harvesting post-senescence means that vegetation is removed after peak biomass and lodging have occurred. In Oklahoma and South Dakota, delaying harvest until October increased yields and decreased N and ash concentrations in CRP biomass compared to pre-peak biomass harvests [16], [23]. Harvesting switchgrass-dominated CRP lands every year compared with alternate years increased yields [24], while deferring harvest to more than two year intervals lowered bioenergy potential in Canadian conservation grasslands managed for wildlife [25].

In the present study, we modeled bioenergy potential of conservation grasslands based on three response variables related to quantity and quality: biomass yield, theoretical ethanol conversion efficiency, and plant tissue N. We used data collected from large-scale plots distributed across three locations of western Minnesota and harvested with commercial-scale tools and techniques. Our objectives were (i) to determine biomass yields, theoretical ethanol conversion efficiency, and plant tissue N content from conservation grasslands, (ii) to measure the variability of bioenergy potential along a latitudinal gradient in western Minnesota, and (iii) to understand what factors affect bioenergy potential by modeling the three response variables with data on plant communities, soil fertility, precipitation, and management activities while accounting for space and time. Two harvest treatments were used to determine if yields from completely harvested plots followed similar trends through time as yields from plots that included previously unharvested regions of biomass. Our results are intended to aid policy and land-management decisions regarding the use of conservation grasslands for bioenergy production in the Upper Midwest, USA.

## Methods

### Experimental design

**Figure 1:**
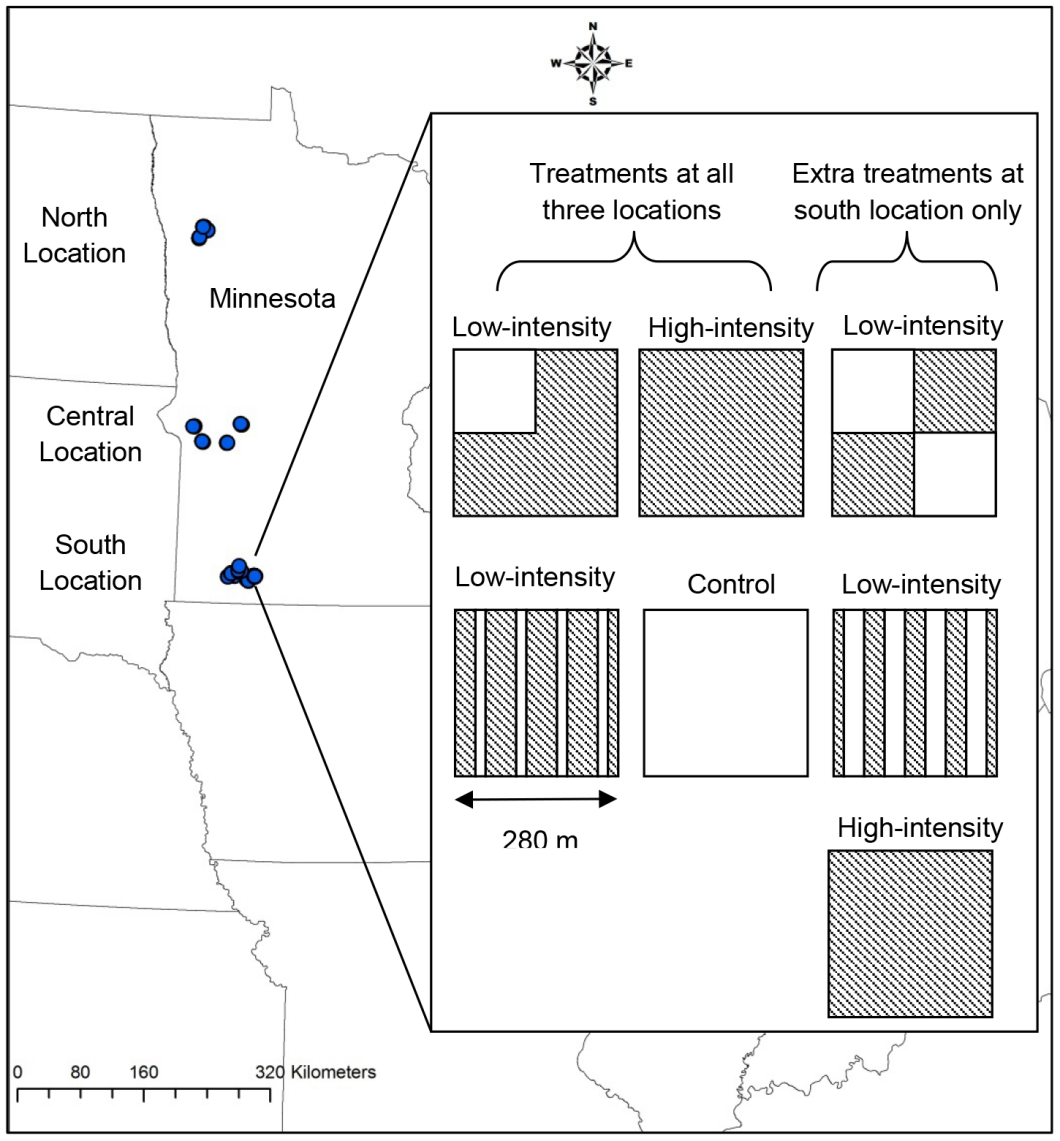
Study areas in Minnesota, located in the Upper Midwest, USA. Research blocks are indicated by circles within the outline of Minnesota in north, central, and south locations. Inset outlines treatments within blocks.

In 2008, we located and delineated 60 plots within existing grasslands enrolled in a conservation program. Plots were distributed among three locations (hereafter north, central, and south locations) spanning a latitudinal gradient in western Minnesota, USA (Figure 1). Soils of the south are glacial till, the north are laucustrine, and the central has regions containing both. Forty plots were located on conservation grasslands managed by the Minnesota Department of Natural Resources (DNR), eight plots managed by the US Fish and Wildlife Service, and 12 plots managed by private landowners as part of the CRP. Each plot was about 8 ha (20 acres; mean = 8.1 ha, SD = 0.5 ha) in size and contained a mixture of grasses and forbs. All plots were established more than five years prior to the project start date. Three of 12 CRP plots were planted with perennial introduced grasses and legumes (CP1) and the rest with perennial native grasses (CP2). The DNR plots were established with different species, but all were categorized as “restored/planted tall grass prairie”. A list of the most frequently observed species is in Table S1. Plots were managed periodically for woody species with prescribed fire and/or mechanical harvest prior to the project start date. Fire was not implemented on our plots during the duration of the study. Occasional spot-spraying of herbicides was done in the south location to control invasive species.

Within each location, treatments were replicated in four blocks (Figure 1). Each block contained a control (no harvest) and three harvested plots. Since the control plots were not harvested, this analysis does not include data from those plots. Plots were randomly assigned a harvest treatment, and, for this analysis, were considered either a high- or low-intensity harvest. High-intensity treatments involved a complete harvest of the assigned plot while low-intensity treatments involved a partial harvest so that the plot contained a refuge of standing vegetation of 2 or 4 ha. The harvest treatments were designed to maintain other uses of the grassland, such as habitat for wildlife. In low-intensity harvest treatments, the refuge moved annually within the fixed plot area so that each year, a portion of the harvested area contained biomass that was not harvested the previous year. At all three locations, each block included one control plot, one high-intensity treatment, and two low-intensity treatments with refuges of 2 ha. A separate sub-study allowed the establishment of extra plots in the south location. Blocks in the south location included one extra high-intensity treatment plot and two extra low-intensity treatment plots (totaling seven plots per block). The extra low-intensity treatment plots had refuges of 4 ha. Twenty four plots were scheduled to be harvested in the south and twelve in each the central and north locations. Weather prevented the harvest of certain plots each year. No plots were harvested in the north in 2011 due to expiring land contracts.

### Field and laboratory methods

A single operator harvested the plots between late October and mid December in 2009, 2010, and 2011. No plots were harvested after the first significant snowfall. Vegetation was harvested to a target height of 15 cm with a self-propelled windrower with a mounted disc cutter. When conditions were deemed dry enough by the operator, the cut biomass was immediately baled using a large round baler. If the cut biomass required drying, it was raked into larger windrows and left to dry before being baled. Due to time constraints and landowner regulations, bales were removed from the plots as soon as possible, therefore individual bales were not weighed from each plot. Instead, bales were loaded onto semi trailers and weighed with a scale certified by the U.S. Department of Transportation on transport for storage. This weight was divided by the number of bales on the trailer to determine an average bale weight and variation (coefficient of variation = 9%; for further details, see Text S1). We divided the sum of all the trailer weights by the total number of bales to generate an overall average bale weight. The average bale weight was multiplied by the number of bales from each plot to estimate total harvested biomass. The perimeter of the cut area in each plot was measured using a hand-held global positioning system (GPS) (Garmin Ltd., Olathe, Kansas, USA) on an all-terrain vehicle. Biomass yield was determined for each plot as the amount of biomass harvested (Mg) divided by the area cut (ha).

While bales were still in the field, core samples were extracted from bales of harvested biomass for each plot with a hay probe (Forageurs Corp., Lakeville, MN, USA) attached to an electric drill. One biomass core was collected from every other bale as they were ejected from the baler; therefore the number of core samples was determined by the size of the harvested area within the plot and biomass productivity (mean number of cores in high-intensity plots = 22). Cores were aggregated by plot and weighed wet immediately after collection (mean sample weight = 156 g), dried at 45° C for four days, reweighed and used here to estimate bale yields on a dry matter basis.

Chemical constituents of the biomass were measured from the aggregated core samples for each plot. Biomass samples were dried at 45° C for four days, ground with a Wiley mill (Thomas-Wiley Mill Co., Philadelphia, PA, USA) to pass a 1 mm screen, and then reground with a cyclone mill. A subsample from each plot was analyzed for N by AgVise Laboratories using methods described on their website (Agvise Inc., Benson MN; http://www.agvise.com).

The concentration of cell wall carbohydrates was determined using near infrared spectroscopy (NIRS) with methods described by Schmer *et al*. [26]. NIRS estimates were from equations built with samples from previous collections, upon which wet chemistry methods were used to directly determine cell wall carbohydrate concentrations (Table S2). The values of xylose, arabinose, mannose, galactose, and glucose were calculated with methods established by the U.S. Department of Energy to predict theoretical ethanol conversion efficiency (Equation S1, http://www1.eere.energy.gov/biomass/ethanol_yield_calculator.html). Calculations used to estimate theoretical ethanol conversion efficiency assume 100% conversion efficiency because realized efficiency rates are not available for production-scale systems.

In the summer of 2009, soil cores were collected to a depth of 20 cm at eight points adjacent to the randomly distributed vegetation quadrats. Soil cores were aggregated by plot and processed and analyzed by AgVise Laboratories for N–NO_3_, pH, organic matter, and cation exchange capacity.

Plant community composition was visually assessed in 1.0×1.5 m quadrats at 12 random points within each plot in late July and/or early August of 2010 and 2011. A total of 24 quadrats were sampled in the high-intensity treatment plots in 2010 to assess sample power. In 2009, plant community data was collected from quadrats, each 0.75×5 m, in all plots. Quadrat locations were generated with ArcGIS 9.3 (ESRI, Redlands, CA, USA) and loaded to hand-held GPS units. Within each quadrat, surveyors identified all plant species and assigned each a score for relative abundance as a percentage of the canopy cover in the quadrat. Bare ground and litter were also assigned a percentage. Species were aggregated into functional groups for analysis. The average cover value for each functional group was calculated by plot.

Cooperative Farming Agreements, Special Use Permits, and a letter of approval were acquired from the Minnesota Department of Natural Resources, US Fish and Wildlife Service, and the US Department of Agriculture Farm Service Agency for permission to conduct research on state, federal and private land.

### Data Analysis

Three response variables related to different components of bioenergy potential were measured in all plots and modeled in this study: biomass yield, theoretical ethanol conversion efficiency, and plant tissue N. Linear mixed effects models were used to test the main effect of location on the three response variables and to determine which covariates were significantly correlated with them. Total variation for each response variable was partitioned into four levels of a temporal/spatial hierarchy that was used as the random structure for the variance components analysis. The largest level of this hierarchy partitioned variance among years, with lower levels partitioning variance between locations, between blocks, and within plots; each level nested within the higher level. A model with only random effects was used to determine the variance at each level of the hierarchical random structure for all three response variables. Equation 1 was modified from West *et al*. [27] to derive variance estimates for each level of the random hierarchy, where *ICC_i_* represents the proportion of variation at level *i* compared with the total variation.
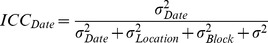


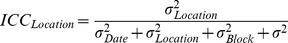


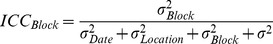



To quantify the differences in biomass yield, ethanol conversion efficiency, and plant N between locations, a dummy variable was assigned to the south, central, and north locations and was modeled as a categorical main fixed effect. Using location as a fixed effect, various random structures composed of the nested spatial/temporal variables were fit to models and compared using maximum likelihood ratio tests.

Land ethanol yield (l ha^−1^) was calculated by multiplying ethanol conversion efficiency (l Mg^−1^) by biomass yield (Mg ha^−1^) for each plot. A linear regression model was used to estimate the fraction of variation in land ethanol yield due to variation in biomass yield.

For each response variable, we selected a group of candidate covariates *a priori* from a list of measured variables (Table 1). A global model for each response variable included all covariates related to plant community structure and an interaction between each community covariate and the main effect of location. No three-way interactions were tested. Each global model included a best fitting random structure and a first order autocorrelation structure. The global model was reduced by removing the least significant fixed effect determined by t-statistic at P<0.05 [28]. This iterative process continued until all fixed effects were removed. The resulting models were compared using Akaike's information criteria adjusted for small sample sizes (AIC_c_) [29]. The best fitting model was refit using restricted maximum likelihood to generate unbiased parameter estimates. For models without interactions, Tukey's *post hoc* means separation test was used to determine differences between levels of significant main effects.

**Table 1:** List and description of all covariates available for analysis.

Effect	Variable	Description
Random	DATE, LOC, BLOCK, PLOT	Nested temporal and spatial variables. Plot nested in block nested in location.
Main	Location	Categorical main effects of location.
Plant Community	C4, C3, Legume, Forb	Continuous measure of mean percent cover of each plant functional group by plot.
Soil Fertility	NO_3_, OM, pH, CEC	Mean values of N–NO_3_ (NO_3_), organic matter (OM), pH, and cation exchange capacity (CEC) by plot.
Plant Composition	PlantN	The concentration of N in harvested biomass tissue.
Precipitation	April, May, June, July, August, September	Total monthly precipitation measured for each year by block.
Interactions	C4×Location, C3×Location, Legume×Location, Forb×Location, Harvest×Location	Interaction between main effects, and between the main effect of location and all plant community covariates

A mixed effect model was used to test the effect of harvest intensity on the change in biomass yield over time. The difference in biomass yield from the first harvest (2009) to the last (2011) was calculated for plots in the south and central locations to test the hypothesis that trends in biomass yields through time would be the same for plots where all the biomass is removed as plots that include regions of previously unharvested biomass. The change in yield was compared between low- and high-intensity harvest treatments. The model included an interaction between harvest intensity and location while accounting for variation in each plot as a random variable. All statistical analyses were conducted with program R [30].

## Results

We analyzed and modeled biomass yield from 109 observations and theoretical ethanol conversion efficiency and plant tissue N from 112 observations from conservation grasslands harvested in autumn of 2009, 2010, and 2011. Weather obstructed biomass harvest at certain plots each year which resulted in an unbalanced data set. No plots were harvested in the north location in 2011 due to expiring land contracts.

The south location received more precipitation during the growing season compared with the north and central locations during all years of the study. Precipitation was lowest in 2009 at the south and central locations, and lowest in 2011 at the north. Over the course of the project, precipitation was the greatest in 2010 and well exceeded the 30-year mean at all locations. In 2011, the north and central locations were below the 30-year mean while precipitation at the central location was higher (Table 2).

**Table 2:** Cumulative precipitation from April through October by location and year, for comparison with other regions.

	2009	2010	2011	30 yr. mean
	(mm)			
**North**	435	663.46	391.51	442.21
**Central**	452.64	663.22	538.59	518.92
**South**	559.09	864.36	577.13	582.93

30 yr mean: http://hurricane.ncdc.noaa.gov/climatenormals/clim81/MNnorm.pdf

Minnesota Climatology Working Group: http://climate.umn.edu/hidradius/HIDENbrowse_PHP.asp

### Biomass yield

**Figure 2:**
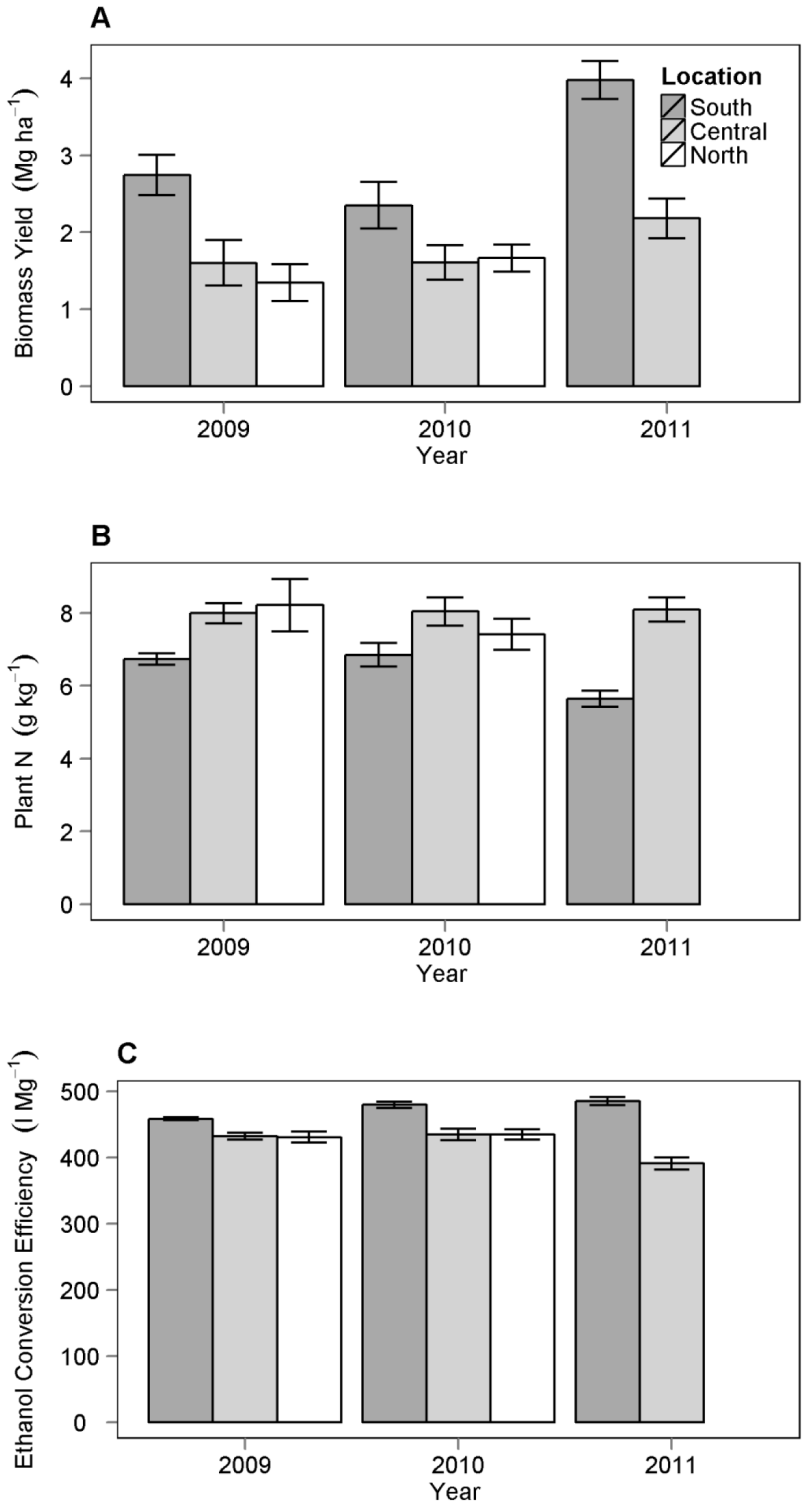
Average values (SE) of response variables by location and year. Mean values of biomass yield (A), plant tissue N (B), and ethanol conversion efficiency (C). Black, gray and white bars are mean values from plots harvested in south, central and north locations respectively.

**Figure 3:**
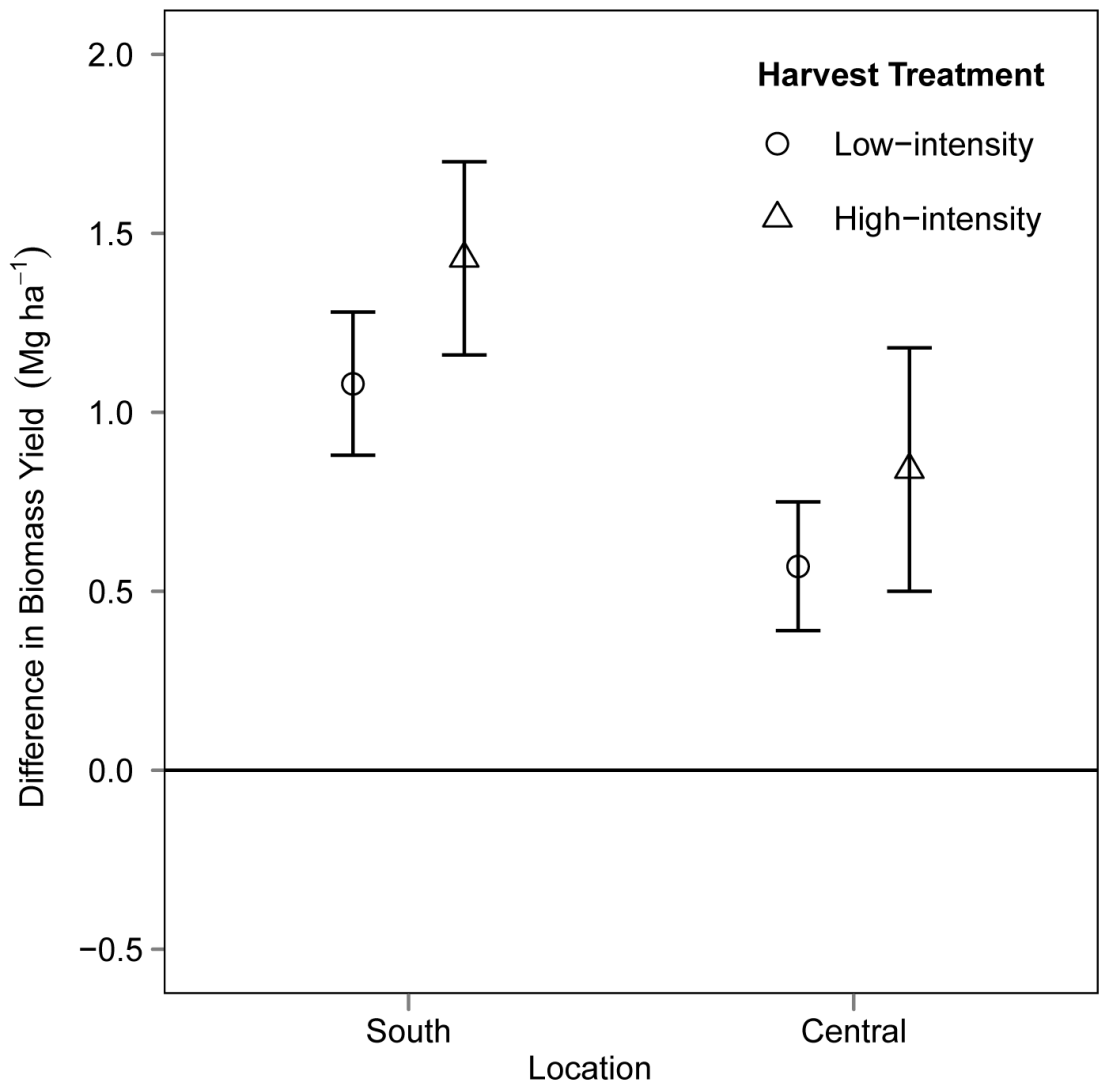
Change in biomass yield from 2009 to 2011 in low- and high-intensity harvest treatments by location. Average change in biomass yield(±90% CI). In low-intensity plots, one third to one half of the annually harvested biomass was from an area not previously harvested. High-intensity harvest plots included biomass from the same area harvested annually.

Without accounting for covariates, mean biomass yield in the south was 55%, 69%, and 55% greater than other locations in 2009, 2010, and 2011 respectively (Figure 2A). Annual plot biomass yield ranged from 0.5 Mg ha^−1^ to 5.7 Mg ha^−1^ and had an overall mean of 2.5 Mg ha^−1^ across all locations and years. Biomass yield increased from 2009 to 2011 in both the south and central locations and in both harvest intensities (Figure 3). The increase in biomass yield through time was the same between harvest intensities (F = 0.48, df = 27, P = 0.49).

### Biomass quality

**Figure 4:**
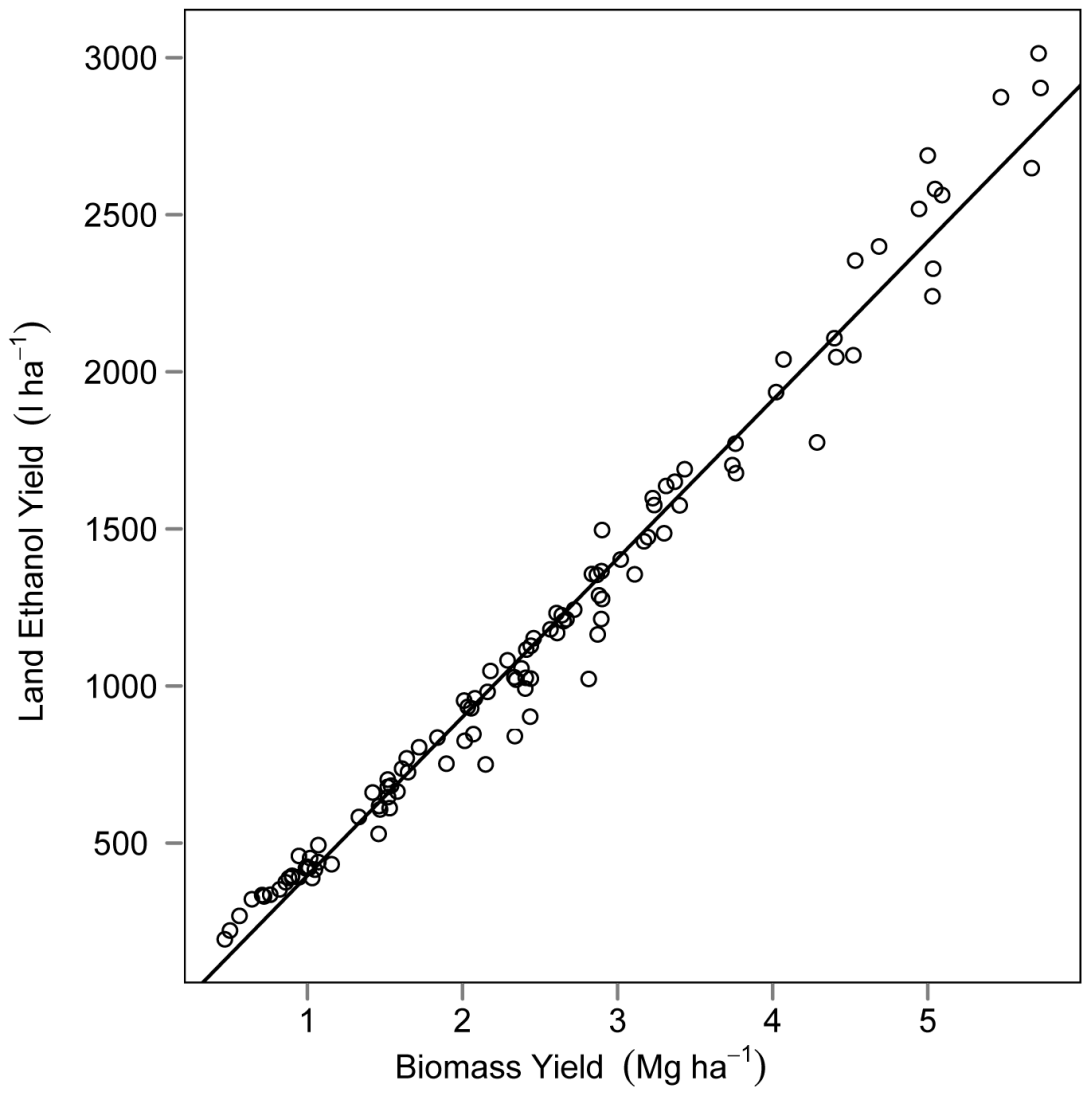
Correlation between land ethanol yield (l ha^−1^) and biomass yield (Mg ha^−1^). Points represent values from conservation grasslands harvested in the autumn of 2009, 2010, and 2011. Regression line from linear model with R-squared value = 0.98.

Biomass yield was a significant predictor of the variation in land ethanol yield (F = 5558, df = 1 and 108, P<0.001). The adjusted R-squared was 0.98 for the relationship between biomass yield and land ethanol yield (Figure 4). Mean ethanol conversion efficiency was 450 l Mg^−1^ with a standard deviation of 38 across all locations and years. Mean plant N concentration was 7.1 g kg^−1^ with a standard deviation of 1.5 and was not consistently different among locations and years. Mean plant N was lower and mean ethanol conversion efficiency was greater in the south than the other locations in all three years (Figure 2B and 2C).

### Variance components analysis

Results from the intercept-only random effects models suggest that of the total variation in biomass yield, ethanol conversion efficiency, and plant N, the variance between years explained the smallest fraction (Table 3). The largest fraction of the variance in biomass yield and plant N was partitioned into within-plot variance, while the variation between locations accounted for about one-third for both responses. More than a majority of variation in ethanol conversion efficiency was observed between locations (Table 3).

**Table 3:** The contribution of variation from nested random effects for measures of bioenergy quantity and quality.

Nested Sources of Variation	Biomass Yield	Ethanol Conversion Efficiency	Plant N
Between years	0.33 (6%)	4.6*10^−3^ (0%)	1.0*10^−4^ (0%)
Between locations	0.74 (31%)	28.78 (57%)	0.86 (34%)
Between blocks	0.65 (24%)	17.45 (21%)	0.15 (1%)
Within plot (residual)	0.82 (39%)	17.85 (22%)	1.18 (65%)

Variation reported as standard deviation and percent of total variation.

### Bioenergy potential models

#### Biomass Yield

**Figure 5:**
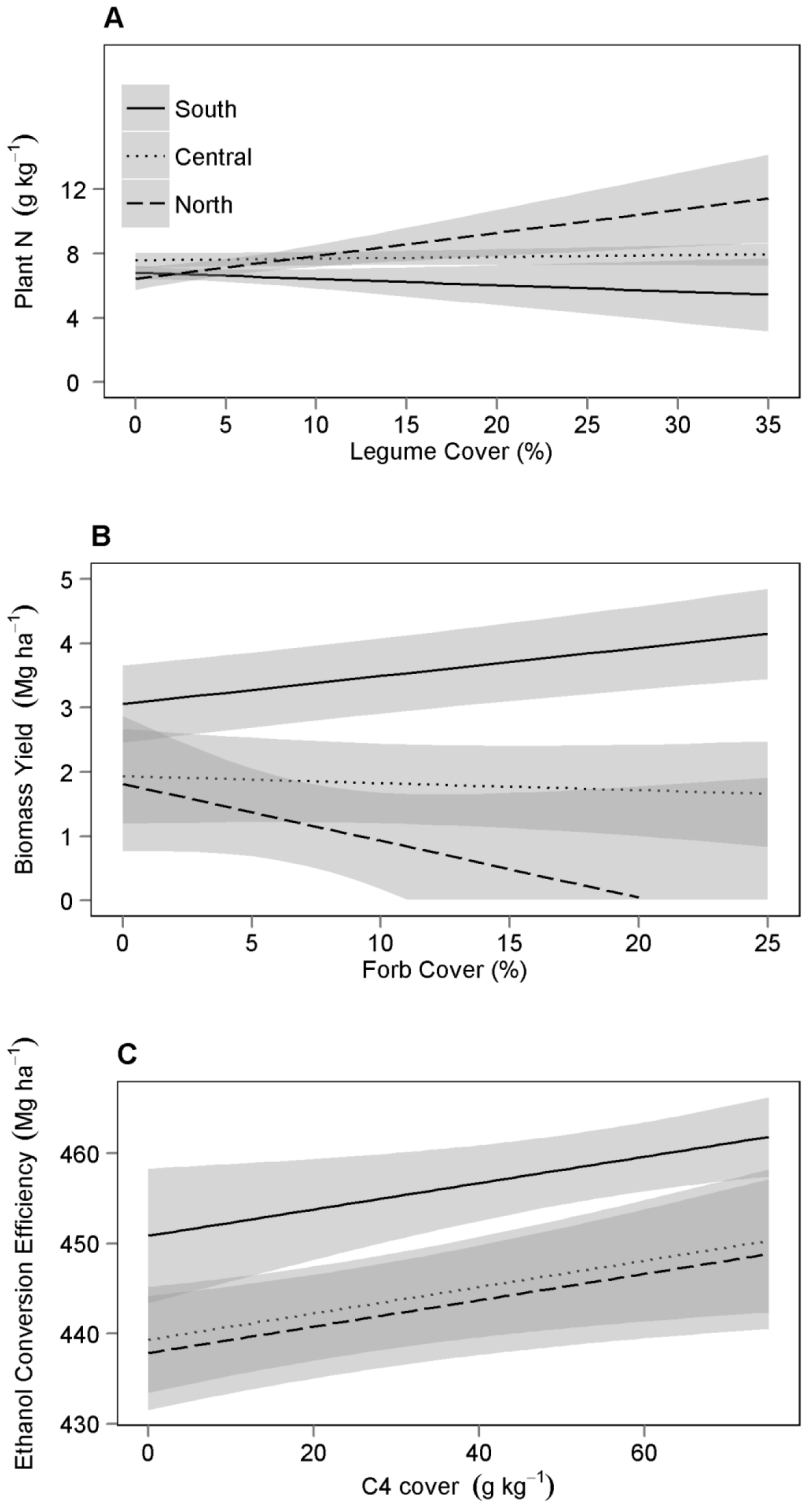
Estimated effect of plant functional group composition on bioenergy potential. Regression line estimates(±90% CI) of the effect of legume cover on the concentration of N in biomass after harvest (A), the effect of forb cover on biomass yield (B), and the effect of C4 cover on ethanol conversion efficiency (C). Estimates are from the best fitting models with all other covariates held constant at their average values.

Measured soil fertility variables did not contribute to explained variation in biomass yield. The effect of forb cover was significant in the best fitting model (Table 4) and influenced biomass yield uniquely in the south compared with the other locations (Table 5, Figure 5B). Specifically, forb cover was negatively correlated with biomass yield in the central and north locations, but positively correlated with biomass yield in the south location. Covariates for May precipitation and legume cover were positively correlated with biomass yield in the best fitting model (Table 5). A model with the random variables plot (identified below as PLOT; see Table 1) nested within block (identified as BLOCK) was superior to a model without random effects (L = 40.77, df = 1, P<0.001). The three best fitting models were similar in their explanatory power determined by AIC_c_ (Table 4).

**Table 4:** Top three best-supported models of bioenergy potential measured from conservation grasslands in Minnesota, USA.

Response	Model	Parameters (K)	ΔAIC_c_
Biomass Yield	Intercept+Location×Forb+May+Legume	12	0.00
	Intercept+Location×Forb+Legume+May+June	13	1.56
	Intercept+Location×Forb+Forb+May	10	2.06
Ethanol conversion efficiency	Intercept+Location+C4+PlantN+Forb	14	0.00
	Intercept+Location+C4+PlantN	13	0.69
	Intercept+Location+C4+Forb+NO3+PlantN	15	1.86
Plant N	Intercept+Location×Legume+C4+NO3	12	0.00
	Intercept+Location×Legume+C4+NO3 +pH	13	0.28
	Intercept+Location+C4+NO3	9	0.42

**Table 5:** Parameter estimates from best-fitted mixed effects models with biomass yield, ethanol conversion efficiency, and plant N as response variables.

Response	Variable	β	SE (β)	DF	t-value	p-value
Biomass Yield	Intercept	2.069	0.381	56	5.432	<0.001
	Location 2	−1.126	0.583	9	−1.932	0.085
	Location 3	−1.243	0.738	9	−1.684	0.126
	May	0.011	0.001	56	9.893	<0.001
	Legume	0.017	0.007	56	2.428	0.018
	Forb	0.044	0.013	56	3.284	0.002
	Location 2×Forb	−0.055	0.026	56	−2.073	0.043
	Location 3×Forb	−0.132	0.076	56	−1.750	0.086
Ethanol Conversion Efficiency	Intercept	529.905	9.680	96	54.743	<0.001
	Location 2	−11.550	4.623	9	−2.498	0.034
	Location 3	−13.005	4.840	9	−2.687	0.025
	C4	0.147	0.070	96	2.081	0.040
	Plant N	−10.812	1.088	96	−9.941	<0.001
	Forb	−0.357	0.203	96	−1.760	0.082
Plant N	Intercept	6.786	0.458	59	14.827	<0.001
	Location 2	0.746	0.400	9	1.862	0.096
	Location 3	−0.384	0.531	9	−0.724	0.488
	C4	−0.017	0.006	59	−2.975	0.004
	Legume	−0.040	0.043	59	−0.925	0.359
	NO3	0.077	0.016	59	4.748	<0.001
	Location2×Legume	0.050	0.044	59	1.137	0.260
	Location3×Legume	0.182	0.071	59	2.579	0.012

#### Ethanol Conversion Efficiency

The two best fitting models included the effect of location, the cover of C4 grass, and the nitrogen content of harvested biomass as predictors of variation in ethanol conversion efficiency. The best fitting model included the cover of forbs and omitted all interactions between main effect and covariates (Table 4). The cover of C4 grass was positively correlated with ethanol conversion efficiency (Figure 5C), while plant N and forb cover showed negative relationships with ethanol conversion efficiency (Table 5). Ethanol conversion efficiency was significantly greater in the south than the central (P = 0.034) and north (P = 0.020) locations, with a metric ton of biomass producing 12% more ethanol in the south than the average of the central and north locations. There was no significant difference between the central and north (P = 0.947) locations. A model with the random variables BLOCK and DATE was best supported for explaining variation in ethanol conversion efficiency. The random structure was fit to allow unique BLOCK variation around the intercept by DATE. This structure was better supported than the fully nested random structure (L = 13.5, df = 1, P = 0.004) and a model without a random structure (L = 64.7, df = 1, P<0.001). The two best fitting models differed by 0.69 AIC_c_ points and one parameter (Table 4).

#### Plant N

The three best fitting models included the main effect of location, C4 cover, and soil N–NO_3_ concentration (Table 4). The best supported model included an interaction term between location and legume cover (Table 5). In the south, legume cover was negatively correlated with plant N as opposed to the positive correlation observed in the central and north locations (Figure 5A). Soil N–NO_3_ and C4 cover were positively and negatively correlated with plant N respectively (Table 5). The best fitting random structure for modeling the concentration of N in biomass included PLOT nested within BLOCK. This structure was superior to a model without a random component (L = 14.9, df = 1, P<0.001) and to a model with a fully nested hierarchy of random variables (L = 9.2, df = 1, P = 0.003).

## Discussion

Harvested biomass yields from low-input grasslands managed for conservation was 2.5 Mg ha^−1^ and on average, fluctuated 23% around this mean across the three year study period. Assuming this yield can be achieved from all the conservation grasslands within an 80 km radius of a biorefinery located in the southwest portion of Minnesota (a total of 107,571 ha of conservation grassland or 5.4% of the total area), and that only 75% of the conservation grasslands are harvestable within that area, approximately 1000 Gw*hours of energy is available (Text S2). If divided across the year, this is equivalent to 114 MW of continuous energy from conservation grasslands alone.

Yields were highest in the south location in all years of this experiment, but were 49% lower than first-year hand-cut yield estimates from newly established high diversity mixtures grown in similar regions [31]. Despite similar growing conditions, the high diversity mixtures were grown on fine loam soil with N, P, and K concentrations more than two times higher than concentrations found in our soils. From our southern plots, biomass yield estimates from hand-cut samples collected in late July were 91% and 54% greater than yield values from commercial-scale harvest in 2010 and 2011 respectively (unpublished data), both of which are similar to the harvest efficiency of managed switchgrass plots in Italy [32]. Although leaf loss and reallocation of C to belowground structures can account for 12% to 19% of decreased biomass yields from September to November [33], there is evidence that commercial-scale harvesting techniques can be made more efficient at both cutting more of the material to a desired height and picking up more of the material with a baler to improve yields [32]. It should be noted that stubble and residual litter provides environmental benefits by reducing erosion and providing cover for ground nesting birds, therefore 100% harvest efficiency may not be a desired objective. Observed variation in litter quantities across studies suggests that caution be taken when comparing aboveground productivity estimates and biomass yields between small-scale and large-scale studies that do not use similar cutting and biomass collection methods.

Generally, the concentration of N in herbaceous biomass results in greater NO_X_ emissions during thermochemical conversion to energy compared with light fuel oil and natural gas [34]. It has been recommended to delay harvesting until after senescence to allow perennial plants to translocate N to belowground tissues for both switchgrass [35] and conservation grassland biomass [16]. Nitrogen content in harvested biomass from this project was similar to conservation grasslands harvested after a killing frost in South Dakota [36]. There is concern that low-input grasslands might not be a long-term viable source of biomass because of N depletion during harvest [37], but those concerns have not yet been tested. There is evidence that long-term annual biomass harvest from low-input grasslands does not decrease yields [38]. Mixed-species grasslands like those used in this project contain legumes that add N annually. N inputs via legumes ranged from 28 to 187 kg ha^−1^ in mowed grass/legume pastures that contained white clover [39], yet studies are needed to determine the net N flux in harvested grassland systems across a range of locations.

**Figure 6:**
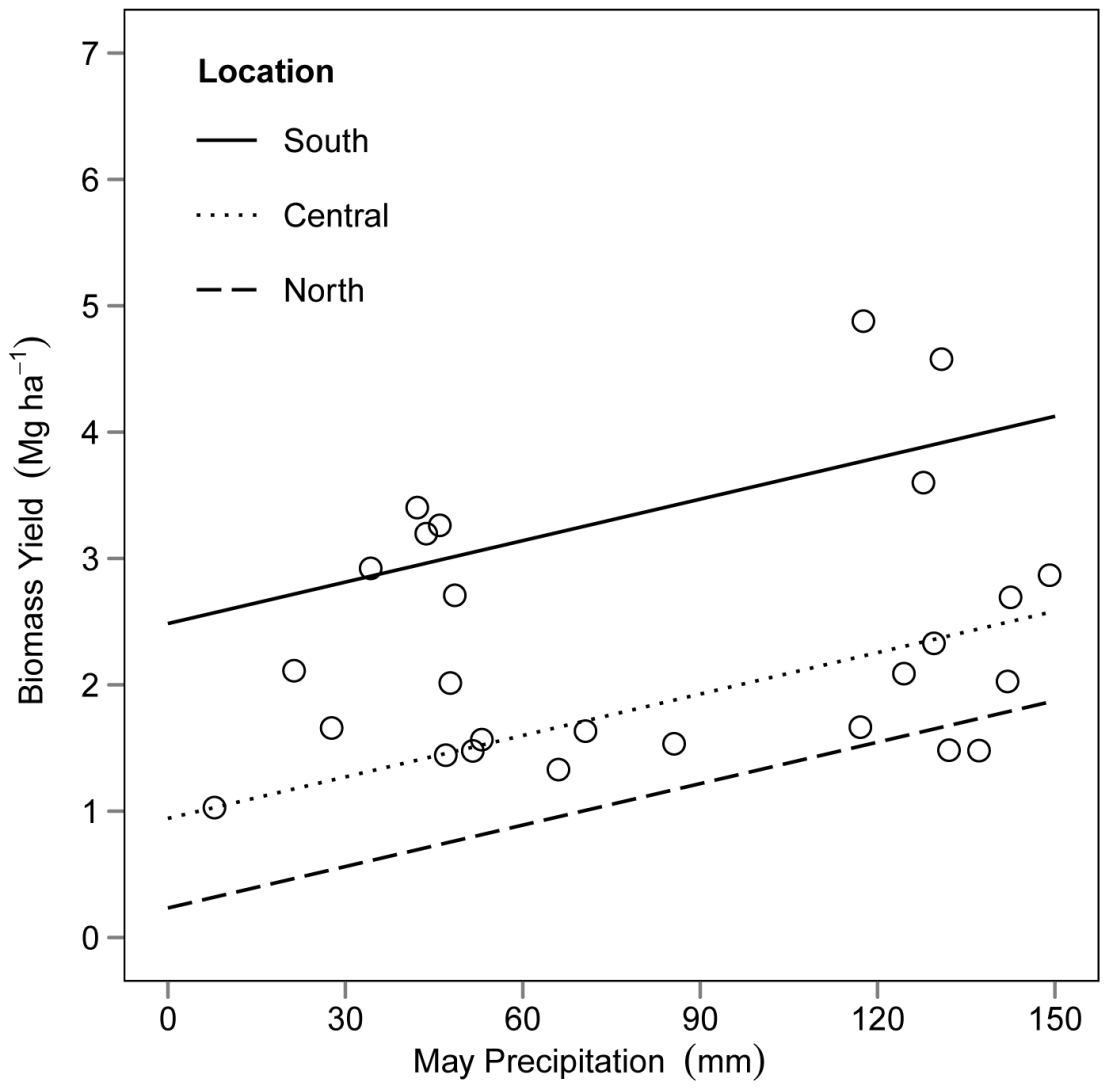
Estimated effect of May precipitation on biomass yield. Dots represent average measured biomass yield and May precipitation values by block. Regression lines are model estimates for bioenergy yield across the precipitation gradient for each location, with all other covariates held constant at their average values.

Variation in biomass yield, ethanol conversion efficiency, and concentration of N in plant tissue was relatively small between years, deviating from each location's average by no more than +/− 27%, 11%, and 7% respectively. This is in contrast to other studies with less mature perennial grasslands (our study sites were all >5years old), where issues with establishment contributed to larger (up to 69%) year-to-year variation in biomass yield [21]. Across the total study area, between-year variability in biomass yield was small despite differences in precipitation. Our results show that precipitation during the month of May measured at the block level is important in determining biomass yield (Figure 6). Total precipitation may not be a good indicator for predicting biomass yields because high amounts of precipitation during harvesting months may result in lower yields due to leaf losses and other inefficiencies in biomass collection, especially when harvesting with production-scale equipment [32]. Excessive precipitation during autumn months inundated some parts of this experiment and prevented the harvest of certain plots each year. Averaged across all years, 83%, 78%, and 74% of the planned harvested areas were harvested in the south, central and north locations respectively. This percentage increased annually in the south and central locations.

Consistent values for biomass quality metrics are important for viable biorefinery production. A substantial fraction of the total variation in biomass yield was observed between locations, which is in accordance with studies on the variation of switchgrass yield [21]. About one-quarter of the total variation in biomass yield was measured between blocks, which was similar to the results of yield variation in C3-dominated grasslands analyzed for bioenergy [20]. Florine *et al*. reported smaller total variation in plant N (SD = 0.4 g kg^−1^) than our results (SD = 1.5 g kg^−1^) [20]. Total variation in ethanol conversion efficiency was relatively small but greater than reports from switchgrass, yet similar in terms of partitioning between spatial and temporal scales [26].

The variation in land ethanol yield was almost exclusively due to variation in biomass yield (Figure 4). Land managers looking to harvest biomass from conservation grassland for ethanol production would maximize revenues by identify high biomass yielding plots as opposed to harvesting plots based on the theoretical ethanol potential of the plants.

We hypothesized that covariates would explain variation among locations (Table 6). However, for all response variables, location remained a significant variable in the best fitting models (Table 5). Best fitting models for biomass yield and plant N included interactions between location and plant community covariates, which provide limited information to draw conclusions as to why differences in these response variables exist across locations. In terms of ethanol conversion efficiency, location was identified as a main source of variation, therefore suggesting that other factors related to space–factors that were not measured in this study–influenced the response.

**Table 6:** Mean values (SD) of covariates by location across all years from conservation grasslands in Minnesota.

Covariate	South	Central	North
		% cover	
C4	56.86 (18.78)	24.94 (18.37)	20.12 (18.71)
C3	18.15 (16.30)	37.77 (19.58)	45.64 (23.15)
Legume	2.80 (3.22)	8.51 (14.57)	4.81 (5.07)
Forb	6.54 (6.57)	10.35 (5.94)	6.26 (3.22)
NO_3_	7.84 (3.94)	11.04 (8.35)	13.76 (12.22)
OM	5.27 (1.33)	6.52 (3.04)	5.38 (1.65)
pH	6.67 (0.49)	7.52 (0.37)	7.68 (0.65)
CEC	22.17 (7.55)	25.66 (7.44)	26.19 (8.08)

Other reports have suggested that plant community characteristics such as C4 grass cover [14] and planted species richness [2] improve biomass yields. In this study, it was the cover of non-legume forbs that explained variation in biomass yield (Table 4 and 5). In the south location, plots with greater average forb cover had higher biomass yields, while in the central and north locations, increasing forb cover was associated with lower yields. We expected, as Adler *et al*. documented, that the cover of C4 grass would be positively correlated with biomass yield, and our competitive models include that variable (Table 4). It is possible that an increase in forb cover displaces C4 grasses, which would explain the negative correlation between forb cover and biomass yield in the central and north locations. The inverse relationship between forb cover and biomass yield in the south could be driven by a high-yielding forb species that is present or abundant in the south but not in the other locations. We explored this possibility and found that common milkweed (*Asclepias syriaca*) was present in 300 sample points in the south and only 50 and 5 sample points in the central and north locations. Using data from all sample points, a Pearson's correlation test showed that the cover of common milkweed was not correlated to the cover of C4 grass (P = 0.303) but was correlated to biomass yield (P = 0.016). This suggests that common milkweed could increase biomass yield without displacing C4 grass cover (Table 6). Other studies have observed increases in forb abundance without associated decreases in biomass production [40].

Harvested areas in the low-intensity harvest treatments included a fraction of the plot where vegetation was left standing the year before. This did not affect biomass yields compared with completely harvested plots. European mixed-species hay yields did not decrease after decades of annual harvest without nutrient inputs [38], though long term studies are needed to verify if similar patterns exist in North American grasslands. The positive correlation of May precipitation with yield could be because it supplies resources before the peak productivity time of C4 grasses, which contribute to biomass yield when harvested in autumn [36]. Other studies have shown that the variation in June soil moisture was positively correlated with C4 grass productivity [41], but soil moisture measurements were not made in our study.

Maximum theoretical ethanol conversion efficiency values were slightly higher than those reported in switchgrass [26] and similar to mixed prairies [42], and were greater in biomass harvested from the south compared with biomass from the central and north locations (Figure 2C). Studies of switchgrass show that harvesting later after plant senescence results in higher potential ethanol conversion efficiency [43], thus a similar pattern could exist in polyculture grasslands. We harvested plots in sequence from the north to the south so that the plants would be at a similar phenological stage at the time of cutting. A negative correlation between plant tissue N and ethanol conversion efficiency was apparent in this study (Table 5), and since plant N decreases with senescence, the later harvest date in the south location may have contributed to higher ethanol conversion efficiency found here. Also, our results confirm previous reports of correlations between C4 grass cover and ethanol conversion efficiency [14] (Figure 5C). In general, C4 grasses have higher levels of fermentable sugars than forbs [44]; therefore ethanol conversion efficiency is expected to decrease with increased forb cover relative to C4 dominated stands. As highlighted in this study, Gillitzer *et al*. showed that the relationship between species composition and biomass yield, rather than species composition and ethanol conversion efficiency, is the more dominant driver of land ethanol yield [42], [45].

Legumes in mixed-species grasslands fix atmospheric nitrogen, which has several consequences for ecosystem functioning including increased productivity [46]. However, in the case of combustion bioenergy, undesirable consequences of legume biomass come in the form of pollution. Legume biomass has relatively higher levels of tissue N than forbs and grasses, which can lead to greater NO_x_ emissions during thermochemical energy conversion [34]. The best fitting model identified a relatively strong trend in legume cover and plant N in the north location (t = 2.579, P = 0.012). Weaker evidence of a relationship was observed in the central (t = 1.137, P = 0.260) and the south locations (t = −0.925, P = 0.359), which could be related to the absence or presence of a specific legume species, as observed in other studies [47]. The estimates from this model predict that a four-fold increase in legume cover (from the observed average of 4.8% to 19.2%) in the north location would increase biomass N concentrations approximately 23%, or to a value of 10.2 g N kg^−1^. Promoting legumes increases functional group diversity, which leads to other ecological benefits including increased soil carbon storage [48]. Also, complementarity among C4 grasses and legumes increases biomass yields [48]. Therefore, we believe that the model-estimated environmental cost of legume abundance in bioenergy grasslands is far outweighed by the ecological and yield benefits they provide.

The three best supported models all suggest that unfertilized soils with naturally higher levels of N–NO_3_ will produce biomass with greater concentrations of tissue N (Table 4). Elevated levels of soil N–NO_3_ could come as a result of N fertilizer, which has been considered as a management tool to increase biomass yields in conservation grasslands [8], [23]. Fertilization experiments show that higher N fertilizer rates lead to higher concentrations of N in biomass tissue for C3-dominated mixed grasslands [49], for switchgrass [50], and other C4 grasses [51]. Nitrogen fertilization can lead to a loss of species and functional group turnover [52], but when fertilized grasslands are harvested, species diversity has been shown to be maintained [53] or increase [40]. When considering N fertilizers, land managers must weigh the potential benefits for biomass yields against potential detrimental effects including undesirable shifts in species composition and decreased biomass quality.

## Conclusions

Biomass quality from mixed-species grasslands not managed for bioenergy is similar to dedicated energy feedstocks, in terms of theoretical ethanol conversion efficiency and biomass N. Almost all of the variation in land ethanol yield is based on biomass yield, therefore efforts should be focused on maximizing biomass yield rather than biomass quality when managing grasslands for land ethanol yield. A combination of climate, soil fertility, and plant community factors influence overall bioenergy potential. The effect of forbs and legumes on biomass yield and tissue N, respectively, were different in the south compared with the central and north locations. The covariates we measured did not explain why theoretical ethanol conversion efficiency was greater in the south compared with the other locations, but the cover of C4 grass was positively correlated with ethanol conversion efficiency. After three continuous years of harvest, leaving a portion of standing biomass within the harvested area does not influence biomass yield of future harvests. Simply focusing on plant community variables to predict bioenergy potential of conservation grasslands across various locations at the scale we studied will not provide accurate estimates; instead attention should be drawn to local variation in soil fertility, climate, and possibly plant species and interactions between these variables.

## Supporting Information

Table S1 Ten most frequently observed species and their average percent cover in sample quadrats.
(DOCX)

Table S2 Calibration statistics for NIRS prediction of forage characteristics and plant cell polysaccharides.
(DOCX)

Equation S1 **Equation to estimate theoretical ethanol conversion efficiency from sugar concentrations.**
(DOCX)

Text S1 **Assessment of bale weight variability for large round bales of biomass harvested from conservation grasslands.**
(DOCX)

Text S2 **Calculations for estimating residential power production from conservation grasslands in SW Minnesota.**
(DOCX)
