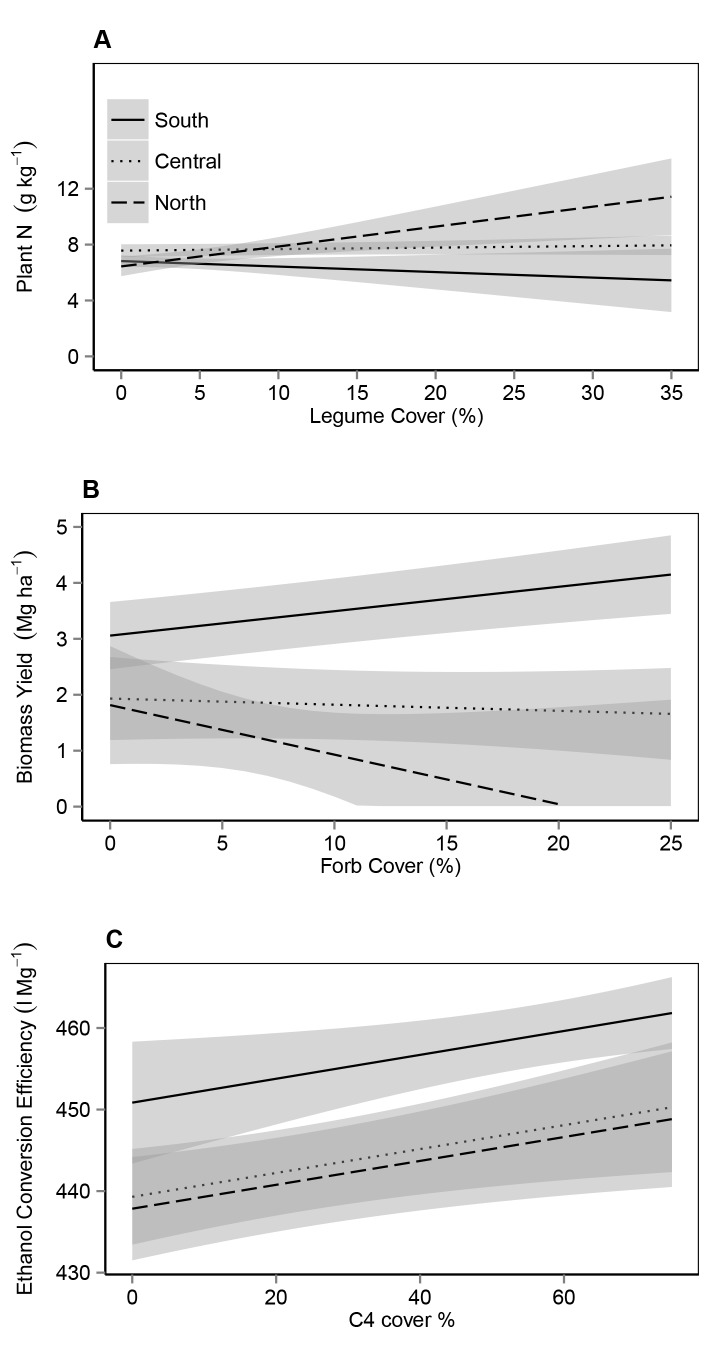# Correction: Energy Potential of Biomass from Conservation Grasslands in Minnesota, USA

**DOI:** 10.1371/annotation/dcd700ef-e870-41d2-bfd1-068aa5b8b717

**Published:** 2013-12-17

**Authors:** Jacob M. Jungers, Joseph E. Fargione, Craig C. Sheaffer, Donald L. Wyse, Clarence Lehman

There was an error in the axis labels of Figure 5. Please see the corrected Figure 5 here: 

**Figure:**