# Supplementary material for: Energy Potential of Biomass from Conservation Grasslands in Minnesota, USA
Source: PLoS One. 2013 Apr 5;8(4):e61209. doi: 10.1371/journal.pone.0061209 (PMC3618185; doi:10.1371/journal.pone.0061209)
Supplement: Table S1 — Ten most frequently observed species and their average percent cover in sample quadrats. (DOCX) [file pone.0061209.s001.docx]

|  | **South** | | **Central** | | **North** | |
| --- | --- | --- | --- | --- | --- | --- |
| **Frequency Ranking** | **Species** | **Average Cover (%)** | **Species** | **Average Cover (%)** | **Species** | **Average Cover (%)** |
| 1 | Andropogon geradii | 34.8 | Poa pratensis | 20.3 | Poa pratensis | 27.2 |
| 2 | Panicum virgatum | 14.6 | Solidago spp. | 8.9 | Solidago spp. | 8.9 |
| 3 | Poa pratensis | 15.6 | Andropogon geradii | 30.4 | Panicum virgatum | 17.9 |
| 4 | Asclepias syriaca | 3.1 | Cirsium arvense | 2.9 | Cirsium arvense | 2.1 |
| 5 | Cirsium arvense | 2.5 | Panicum virgatum | 10.6 | Andropogon geradii | 38.9 |
| 6 | Bromus inermis | 25.2 | Phalaris arundinacea | 33.3 | Phleum pratense | 4.8 |
| 7 | Schizachyrium scoparium | 11.8 | Bromus inermis | 23.4 | Taraxacum officinale | 1.5 |
| 8 | Solidago spp. | 7.1 | Sonchus oleraceus | 4.3 | Sporobolus heterolepis | 22.9 |
| 9 | Melilotus alba | 14.1 | Schizachyrium scoparium | 24.7 | Dalea purpurea | 3.5 |
| 10 | Elymus canadensis | 8.2 | Melilotus alba | 12.2 | Agropyron repens | 9.6 |
