# Supplementary material for: Energy Potential of Biomass from Conservation Grasslands in Minnesota, USA
Source: PLoS One. 2013 Apr 5;8(4):e61209. doi: 10.1371/journal.pone.0061209 (PMC3618185; doi:10.1371/journal.pone.0061209)
Supplement: Table S2 — Calibration statistics for NIRS prediction of forage characteristics and plant cell polysaccharides. (DOCX) [file pone.0061209.s002.docx]

| Perten Only | NDF | IVTD | Klausen Lignin | Rhamnose | Arabinose | Xylose | Mannose | Galactose | Glucose |
| --- | --- | --- | --- | --- | --- | --- | --- | --- | --- |
|  |  |  | -----------------------------------------------------mg/g------------------------------------------ | | | | | | |
| Factors | 7 | 8 | 13 | 7 | 6 | 8 | 12 | 7 | 8 |
| SEC | 1.18 | 1.54 | 6.56 | 1.33 | 3.74 | 19.70 | 1.60 | 1.21 | 13.83 |
| SECV | 2.15 | 1.85 | 11.02 | 1.55 | 4.23 | 22.87 | 2.33 | 1.51 | 16.34 |
| R | 0.885 | 0.906 | 0.783 | 0.862 | 0.763 | 0.895 | 0.916 | 0.907 | 0.927 |
| Range | 63.5 – 81.6% | 31.8 – 49.4% | 153 – 220 | 1 – 12 | 14 – 40 | 45 – 203 | 1 – 25 | 4 – 21 | 185 – 378 |
| N | 76 | 66 | 66 | 73 | 72 | 78 | 75 | 70 | 77 |
|  |  |  |  |  |  |  |  |  |  |
| Perten + Historical Foss |  |  |  |  |  |  |  |  |  |
| Factors | 7 | 8 | 9 | 6 | 10 | 5 | 8 | 6 | 4 |
| SEC | 2.07 | 1.82 | 11.51 | 1.6 | 3.88 | 27.70 | 2.78 | 2.30 | 24.67 |
| SECV | 2.18 | 2.07 | 12.48 | 1.51 | 3.59 | 20.79 | 2.52 | 2.09 | 21.29 |
| R | 0.864 | 0.891 | 0.652 | 0.885 | 0.825 | 0.872 | 0.898 | 0.844 | 0.871 |
| Range | 63.5 – 81.6% | 31.8 – 49.7% | 153 – 260 | 1 – 12 | 12 – 43 | 45 – 242 | 1 – 25 | 4 – 27 | 185 – 424 |
| N | 123 | 107 | 374 | 394 | 373 | 383 | 397 | 407 | 377 |
