# Supplementary material for: Energy Potential of Biomass from Conservation Grasslands in Minnesota, USA
Source: PLoS One. 2013 Apr 5;8(4):e61209. doi: 10.1371/journal.pone.0061209 (PMC3618185; doi:10.1371/journal.pone.0061209)
Supplement: Equation S1 — Equation to estimate theoretical ethanol conversion efficiency from sugar concentrations. (DOCX) [file pone.0061209.s003.docx]

((( glucan + galactan + mannan ) * 172.82 ) + (( xylan + arabinan ) * 176.87 )) * 0.01
