# Supplementary material for: Energy Potential of Biomass from Conservation Grasslands in Minnesota, USA
Source: PLoS One. 2013 Apr 5;8(4):e61209. doi: 10.1371/journal.pone.0061209 (PMC3618185; doi:10.1371/journal.pone.0061209)
Supplement: Text S1 — Assessment of bale weight variability for large round bales of biomass harvested from conservation grasslands. (DOCX) [file pone.0061209.s004.docx]

Using the information from multiple trailer loads, an assessment of variability was measured. The standard deviation of average bale weights from 13 trailer loads in 2010 was 45 kg. This was similar to published variance values of large round bales of switchgrass (sd = 36 kg; Monti et al., 2009).
