# Supplementary material for: Energy Potential of Biomass from Conservation Grasslands in Minnesota, USA
Source: PLoS One. 2013 Apr 5;8(4):e61209. doi: 10.1371/journal.pone.0061209 (PMC3618185; doi:10.1371/journal.pone.0061209)
Supplement: Text S2 — Calculations for estimating residential power production from conservation grasslands in SW Minnesota. (DOCX) [file pone.0061209.s005.docx]

Total CRP in SW 80 mile radius = 185626 acres, WMA = 66337, WPA = 13853; SUM = 265816 * 0.75 = 199362 acres = 80678 ha

80678 ha * 2.5 Mg / ha = 201695 Mg

201695 Mg * 18.5 GJ / Mg = 3731357 GJ

3731357 GJ * 0.278 MW*h = 1037317 MW*h

Average U.S. household electricity consumption = 11.5 MW*h/year

1037317 MW*h / 11.5 MW*h/house = 90201 homes
